# Plasma Levels of Matrix Metalloproteinase (MMP)-2, MMP-9 and Tumor Necrosis Factor-α in Chronic Hepatitis C Virus Patients

**DOI:** 10.2174/1874285801509010136

**Published:** 2015-08-31

**Authors:** Mohamed S Abdel-Latif

**Affiliations:** Department of Medical Laboratory Technology, Faculty of Allied Medical Science, Pharos University in Alexandria, Egypt

**Keywords:** Metalloproteinase (MMP)-9, MMP-2, TNF-α, liver ﬁrbrosis, HCV

## Abstract

**Background:**

In chronic HCV infection, pathological accumulation of the extracellular matrix is the main feature of liver fibrosis; that indicates the imbalanced rate of increased matrix synthesis to decreased breakdown of connective tissue proteins. Matrix metalloproteinases (MMPs) play a crucial role in remodeling of extracellular matrix. It is known that expression of MMPs is regulated by Tumor necrosis factor (TNF)-α. Also, levels of TNF-α in liver and serum are increased in chronic HCV patient. Accordingly, this study aimed to correlate the plasma levels of MMP-2, MMP-9 and TNF-α in chronic HCV patients with the pathogenesis of the liver.

**Methods:**

The current study was conducted on 15 ﬁbrotic liver cases with detectable HCV RNA, 10 HCV cirrhotic liver cases, and 15 control subjects of matched age and sex. Plasma MMP-2, MMP-9 and TNF-α were measured by ELISA.

**Results:**

Data revealed that the MMP2, MMP9 and TNF-α levels showed a significant elevation in chronic HCV patients compared to control group (p= 0.001). But, no significant correlation was observed in levels of MMP-2, MMP-9, and TNF-α between fibrotic and cirrhotic cases.

**Conclusions:**

MMP-2, MMP-9 and TNF-α showed high reproducibility to differentiate chronic HCV patients from control group. On the contrary, MMP-2, MMP-9 and TNF-α were not able to differentiate fibrotic from cirrhotic liver cases. Thus, MMP-2, MMP-9 and TNF-α could not be correlated with the progression of liver disease. Rather they could be used as prognostic markers of liver fibrosis.

## INTRODUCTION

Chronic HCV patients are susceptible to hepatic inflammation, developing liver fibrosis, cirrhosis and hepatocellular carcinoma (HCC). Such a case does not depend on virus genotype or viral load [1].

In the liver, pathological accumulation of the extracellular matrix (ECM) is the main feature of fibrogenesis; that indicates the imbalanced rate of increased matrix synthesis to decreased breakdown of connective tissue proteins which is regulated by the matrix metalloproteinases (MMPs). Apparently, MMPs play a central role in remodeling of extracellular matrix and are involved in fibrogenesis and carcinogenesis [2]. Remodeling of ECM is associated with regulation of different levels of MMPs, which is mediated by regulated MMPs gene expression, activation of MMPs, and tissue inhibitor of metalloproteinases (TIMPs) [3]. Recently, it has been shown that the increased levels of hepatic gelatinases, such as MMP-2 and MMP-9, are associated with the fibrotic index in chronic HCV patients and with progression and recurrence of HCC [4, 5].

HCV infection stimulates the production of inflammatory cytokines and chemokines; also enhances the enrollment of inflammatory cells to the liver, that resulting in hepatic inflammation and chronic hepatitis [6-8].

In addition, levels of tumor necrosis factor (TNF)-α are known to be increased in liver and serum of chronic HCV patients [9, 10]. Subsequently, TNF-α elevation, which may interfere with insulin signaling, could be the key molecule of processes like inflammation, steatosis, and fibrosis in chronic HCV patients [11].

The aim of this study was to correlate the plasma levels of MMP-2, MMP-9 and TNF-α in chronic HCV patients with the pathogenesis of liver.

## SUBJECTS AND METHODS

### Subjects

This study was conducted on 25 patients with chronic HCV infection and 15 healthy individuals of matched age and sex. Patients and healthy individuals were recruited from the Gastro-enterology department, Medical Research Institute, Alexandria University, and divided into three groups. Group one (fibrotic liver cases) included 15 patients with detectable HCV RNA who were referred to the hepatology unit having liver fibrosis as evaluated by liver biopsy. Group two (cirrhotic liver cases) included 10 patients with established liver cirrhosis as diagnosed by clinical, laboratory, and sonographic data. All were selected to have advanced degree of liver fibrosis as detected histopathologically using the Metavir scoring system (F1-F4). Group three (control = F0) included 15 healthy individuals with normal liver function tests, normal complete blood picture, no HCV and/or HBV infection, and normal abdominal ultrasound examination.

Group one and two met the following inclusion criteria: no diabetes mellitus, no co- infection with HBV, no evidence of hepatocellular carcinoma and no schistosomiasis. Also, they were seropositive for antibodies to HCV genotype 4 as detected by ELISA technique and HCV genotyping, positive for HCV RNA by PCR and did not receive any interferon therapy. Ethical approval for the study was obtained from the local ethical committee of the Medical Research Institute.

All individuals, after obtaining an informed consent, were subjected to routine laboratory investigation, ultrasound examination and percutaneous ultrasound-assisted needle liver biopsy; similarly, as described in our previously published data [12].

### Assessment of Plasma MMP-2, MMP-9 and TNF-α Levels

Enzyme linked immune-sorbent assay (ELISA) kits (RayBio^®^ Human, USA) were used to evaluate plasma levels of MMP-2, MMP-9 and TNF-α according to manufacturer recommendation.

### Statistical Analysis

Data obtained in the present study were statistically evaluated using SPSS version 15 (SPSS Inc., Chicago, IL, USA.). Numerical data were expressed as mean ± SD and the analyses were considered statistically significant at a two-sided *P*≤0.05. Receiver operating characteristic (ROC) analysis was performed to determine the diagnostic significance of MMP-2, MMP-9 and TNF-α. ROC curve was plotted to analyze recommended cut-off values for MMP-2, MMP-9 and TNF-α; also to differentiate between F1–F4 and F0. The area under the ROC curve (AUC) denotes the diagnostic performance of the marker. Agreement of this cut-off value for MMP-2, MMP-9 and TNF-α was expressed in sensitivity, specificity, positive predictive value, negative predictive value and accuracy. Significance of the test results is quoted as two-tailed probabilities, using student *t*-test.

## RESULTS

Results that obtained from routine laboratory investigation, ultrasound examination and percutaneous ultrasound-assisted needle liver biopsy were clearly shown in our previously published study [12].

MMP-2, MMP-9 and TNF-α levels were measured in all plasma samples, from control subjects and chronic HCV patients, using ELISA technique.

### Assessment of Plasma MMP-2

**Fig. (1):**
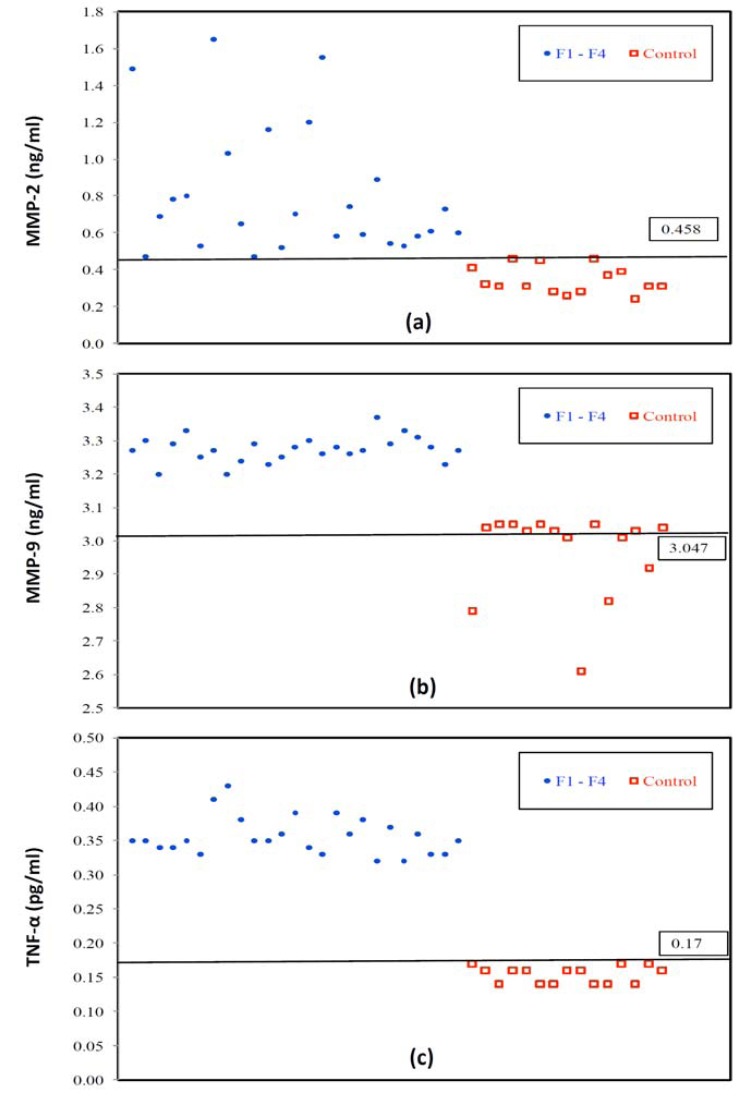
The distribution values of (a) MMP-2, (b) MMP-9 and (c) TNF-α levels around the cut-off. F1-F4 = chronic HCV patients, and F0 = healthy control, according to Metavir score.

Plasma levels of MMP-2 in group three (control subjects) ranged from 0.24 – 0.46 ng/mL with mean value of 0.34 ± 0.07 ng/mL. In chronic HCV patients (group one and group two), plasma levels of MMP-2 ranged from 0.41 – 1.65 ng/mL with mean value of 0.80 ± 0.35 ng/mL. Mean level of plasma MMP-2 was significantly higher in chronic HCV patients when compared to the control group, (p<0.001). Consequently, a positive significant correlation of MMP-2 levels was observed for discriminating chronic HCV patients (Metavir score F1-F4, as revealed by liver biopsy) from control group (F0) (Fig. **1a**). On the contrary, levels of MMP-2 in fibrotic and that in cirrhotic cases were not significantly correlated (p=0.166; data not shown). Therefore, MMP-2 could not be used in differentiating fibrotic cases (F1-F3) from cirrhotic cases (F4).

### Assessment of Plasma MMP-9

Plasma levels of MMP-9 in group three (control subjects) ranged from 2.61 – 3.05 ng/mL with mean value of 2.97 ± 0.13 ng/mL. In chronic HCV patients (group one and group two), plasma levels of MMP-9 ranged from 3.20 – 3.37 ng/mL with mean value of 3.27 ± 0.04 ng/mL. Mean level of plasma MMP-9 was significantly higher in chronic HCV patients when compared to the control group, (p<0.001). Consequently, a positive significant correlation of MMP-9 levels was observed for discriminating chronic HCV patients (Metavir score F1-F4, as revealed by liver biopsy) from control group (F0) (Fig. **1b**). On the contrary, levels of MMP-9 in fibrotic and that in cirrhotic cases were not significantly correlated (p=0.157; data not shown). Therefore, MMP-9 could not be used in differentiating fibrotic cases (F1-F3) from cirrhotic cases (F4).

### Assessment of Plasma TNF-α

Plasma levels of TNF-α in group three (control subjects) ranged from 0.14 – 0.17 pg/mL with mean value of 0.15 ± 0.01 pg/mL. In chronic HCV patients (group one and group two), plasma levels of TNF-α ranged from 0.32 – 0.43 pg/mL with mean value of 0.36 ± 0.03 pg/mL. Mean level of plasma TNF-α was significantly higher in chronic HCV patients when compared to the control group, (p<0.001). Consequently, a positive significant correlation of TNF-α levels was observed for discriminating chronic HCV patients (Metavir score F1-F4, as revealed by liver biopsy) from control group (F0) (Fig. **1c**). On the contrary, levels of TNF-α in fibrotic and that in cirrhotic cases were not significantly correlated (p=0.760; data not shown). Therefore, TNF-α could not be used in differentiating fibrotic cases (F1-F3) from cirrhotic cases (F4).

ROC curve analysis was performed to determine the diagnostic significance of MMP-2 and MMP-9 compared to TNF-α (Fig. (**2a**), Table **1**). The sensitivity, specificity, and accuracy of MMP-2, MMP-9 and TNF-α for discriminating control group (F0) from chronic HCV patients (F1-F4) were 100% each. The area under ROC curve (AUC) was 1.000 each (p<0.001). However, the closer the AUC value of one, the better the overall diagnostic performance of the test. On the other hand MMP-2, MMP-9 and TNF-α were failed to discriminate fibrotic cases (group one, F1-F3) from cirrhotic cases (group two, F4) (Fig. (**2b**), Table **2**).

## DISCUSSION

In liver diseases, determining the stage of fibrosis is of great importance for improved diagnosis, prognosis and administration of the effective therapy [13].

The potential role of MMP-2 for prognosis of liver fibrosis remains unclear, due to contradictory data was reported in different studies [14, 15]. On the contrary, it has been shown that MMP-9 has a great value in the diagnosis of hepatocellular carcinoma; However, a study showed that MMP-9 levels were not significantly correlated to the fibrotic index of the liver in chronic HCV patients [16, 17].

Also, TNF-α appears to play a key role in the necroinflammatory processes occurring in the liver of chronic HCV patients. An independent association has been noted between hepatic inflammatory activity and the level of TNF-mRNA. A positive significant correlation has been shown between serum TNF-α levels and the severity of hepatic inflammation in some but not all cases [18].

The present study was designated to correlate the levels of MMP-2, MMP-9 and TNF-α with the pathogenesis of chronic liver diseases (CLD) caused by HCV, it also aimed at using them as possible non-invasive serum markers for liver fibrosis. Accordingly, we measured the levels of MMP2, MMP9 and TNF-α in 40 cases (25 chronic HCV patients and 15 healthy control subjects).

Demographic and clinical laboratory data of the studied cases were in accordance with the natural history of HCV [12].

MMP2, MMP9 and TNF-α showed a significant elevation in patients with liver fibrosis (F1–F4) compared to control group (p=0.001). Area under ROC curve for MMP2, MMP9 and TNF-α, to discriminate chronic HCV patients (F1–F4) from control group (F0), was 1.000 each (p=0.001). Recently, it has been shown that serum MMP2 concentrations were significantly higher in stage F2-F4 patients compared to controls [19]. Area under ROC curve was 0.770; which is in accordance with our obtained data.

Meanwhile, obtained results showed that, there was no significant correlation between the METAVIR score and levels of MMP2, MMP9 and TNF-α (P values: 0.166, 0.157 and 0.760; respectively) in fibrotic cases and cirrhotic cases. The area under the ROC curve of MMP2, MMP9 and TNF-α for discriminating fibrotic cases (F1-F3) from cirrhotic cases (F4) were 0.667, 0.670 and 0.537 respectively. In such a manner, obtained data were in agreement with other studies that reported this insignificant correlation [20-22].

According to current data, MMP-9 mean serum levels were significantly higher in chronic HCV patients than in controls (p<0.05), which comes in agreement with what has been published [22]. But others reported that serum MMP-9 was significantly lower in patients with chronic HCV than in controls [21]. This variability in results may be related to differences in study populations, type and severity of CLD and difference in assay systems. 

Also, it has been shown that TNF-α, is significantly higher in cirrhotic patients compared to chronic HCV patients with no or mild fibrosis [23]. On the contrary, current data revealed that TNF-α level is significantly higher in both fibrotic and cirrhotic cases than in healthy control subjects.

In conclusion, MMP2, MMP9 and TNF-α showed high reproducibility to differentiate patients with liver fibrosis (F1–F4) from control group. But, MMP2, MMP9 and TNF-α were not able to differentiate fibrotic liver cases (F1–F3) from cirrhotic liver cases (F4). Thus current results suggested that MMP2, MMP9 and TNF-α could be considered as potential markers of inflammation but not of the degree of liver fibrosis in chronic HCV patients.

## Figures and Tables

**Fig. (2):**
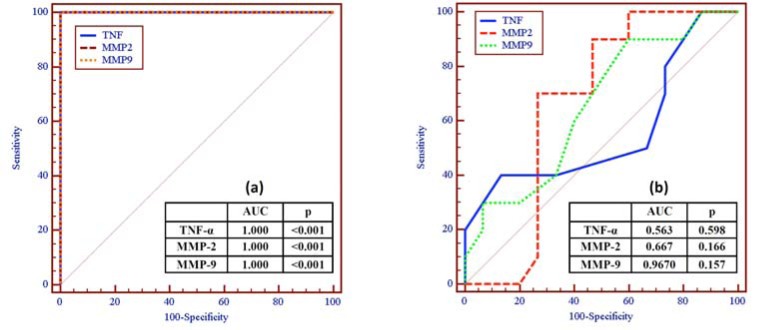
ROC curve of MMP-2, MMP-9 and TNF-α levels for discriminating: (a) chronic HCV patients (F1-F4) from healthy control (F0), and (b) fibrotic cases (F1-F3) from cirrhotic cases (F4).

**Table 1.** Sensitivity, specificity and accuracy for TNF-α, MMP-2 and MMP-9 in control (F0) and chronic HCV patients (F1-F4).

	Cut-off	F0 (No. of cases)	F1-F4 (No. of cases)	AUC	p value	Sensitivity	Specificity	PPV	NPV	Accuracy
TNF-α	≤0.17	15	0	1.000*	<0.001	100.0	100.0	100.0	100.0	100.0
	>0.17	0	25							
MMP-2	≤0.46	15	0	1.000*	<0.001	100.0	100.0	100.0	100.0	100.0
	>0.46	0	25							
MMP-9	≤3.05	15	0	1.000*	<0.001	100.0	100.0	100.0	100.0	100.0
	>3.05	0	25							

AUC, area under the curve; PPV, positive predictive value; NPV, negative predictive value.

* AUC closer to 1 means higher diagnostic performance.

**Table 2.** Sensitivity, specificity and accuracy for TNF-α, MMP-2 and MMP-9 in fibrotic cases (F1-F3) and cirrhotic cases (F4).

	Cut-off	F1-F3 (No. of cases)	F4 (No. of cases)	AUC	p	Sensitivity	Specificity	PPV	NPV	Accuracy
TNF-α	>0.33	13	6	0.563*	0.598	40.0	86.67	66.67	68.42	68.0
	≤0.33	2	4							
MMP-2	>0.74	8	2	0.667*	0.166	80.0	53.33	53.33	80.0	64.0
	≤0.74	7	8							
MMP-9	≤3.25	4	1	0.670*	0.157	90.0	26.67	45.0	80.0	52.0
	>3.25	11	9							

AUC, area under the curve; PPV, positive predictive value; NPV, negative predictive value.

* AUC closer to 1 means higher diagnostic performance.
